# Teachers’ views on the acceptability and implementation of the Incredible Years^®^ Teacher Classroom Management programme in English (UK) primary schools from the STARS trial

**DOI:** 10.1111/bjep.12493

**Published:** 2022-03-11

**Authors:** Kate Allen, Lorraine Hansford, Rachel Hayes, Bryony Longdon, Matthew Allwood, Anna Price, Sarah Byford, Brahm Norwich, Tamsin Ford

**Affiliations:** ^1^ College of Medicine and Health University of Exeter UK; ^2^ Nuffield Department of Primary Care Health Sciences University of Oxford UK; ^3^ King's Health Economics, Institute of Psychiatry, Psychology & Neuroscience King’s College London UK; ^4^ Graduate School of Education University of Exeter UK; ^5^ Department of Psychiatry University of Cambridge UK

**Keywords:** acceptability, implementation, incredible years, primary school, qualitative, teacher classroom management

## Abstract

**Background:**

The Incredible Years^®^ (IY) Teacher Classroom Management (TCM) programme may reduce disruptive behaviour in the classroom and improve child and teacher mental health; however, few studies have considered how acceptable TCM is to teachers or what facilitators and barriers there are to its implementation.

**Aims:**

In this paper we examine the acceptability of the full 6‐day TCM programme and teachers’ perceived barriers and facilitators to implementation in the English (UK) primary school context.

**Sample:**

Forty‐four English (UK) primary school teachers who attended the TCM programme as part of the STARS trial.

**Methods:**

We completed focus groups and telephone interviews with participating teachers 2 months after they completed the TCM programme. Thematic analysis was used to examine the data, and a framework approach was applied to organize and summarize themes.

**Results:**

Teachers liked the structure of the course, the peer group learning environment, delivery methods, and the opportunity to reflect outside the classroom on their practice. They reported that the video clips used lacked cultural relevance and highlighted the importance of group leadership style. Perceived facilitators to implementation included an understanding of the theory underpinning TCM and adaptability of the TCM strategies. Barriers included perceived gaps in the course content in relation to challenging behaviour, applying strategies with older children and the school context within which teachers were working.

**Conclusion:**

Our findings suggest high levels of acceptability to TCM overall, but also highlight the need for a whole school approach to combat potential barriers to implementation.

## Background

Disruptive behaviour was highlighted by The Office for Standards in Education (Ofsted (2019) as one of the main sources of stress for teachers. It impacts negatively on well‐being (Beltman, Mansfield, & Price, 2011; Jennings & Greenberg, 2009; Ofsted, 2019), contributes to poor and worsening teacher retention rates as reported in the UK (see Industrial Injuries Advisory Education Support Partnership, 2018; The Industrial Injuries Advisory Council, 2017), and reduces opportunities for children to learn and thrive (Ofsted, 2019). Despite this, teachers report that they lack adequate support and training to effectively manage the disruptive behaviour in their classrooms (Ofsted, 2019).

The Incredible Years^®^ (IY) Teacher Classroom Management (TCM) programme (Webster‐Stratton, Reinke, Herman, & Newcomer, 2011) may be one way to support teachers in reducing disruptive behaviour in the classroom. Designed in Seattle as one element of a suite of three programmes aiming to reduce disruptive behaviour and promote socio‐emotional development among children, TCM draws on Patterson’s theories of coercive cycles of interaction between adults and children (Patterson, 1982), Bandura’s ideas about modelling and self‐efficacy (Bandura, 1977) and Piaget’s developmental interactive learning methods (Piaget & Inhelder, 1962) to enhance teachers’ behaviour management skills. The programme has four specific goals: (1) to enhance teacher behaviour management skills and improve teacher‐pupil relationships; (2) to assist teachers to develop effective individual and group behaviour plans in order to enable proactive (instead of reactive) classroom management; (3) to encourage teachers to adopt and promote social and emotional regulation skills; and (4) to encourage teachers to strengthen positive teacher‐parent relationships (see https://incredibleyears.com/programs/teacher/classroom‐mgt‐curriculum/ for more details). The programme is delivered to teachers through six whole day sessions spread over a period of 6 months to allow teachers time to experiment with novel strategies and for new behaviours to become embedded into their practice. The sessions involve reflecting on practice, group problem solving, modelling and practicing strategies, group discussions, and role play (Webster‐Stratton et al., 2011).

Evaluations of TCM when delivered in isolation from the parent and child focused IY programmes across the world have demonstrated reduced use of negative teacher classroom management strategies (e.g. use of warnings, negative physical behaviour, criticism, unlabelled praise), greater use of positive teacher classroom management strategies (e.g. use of positive affect, encouragement, labelled praise), and fewer child conduct problems (Chuang, Reinke, & Herman, 2020; Nye, 2017). More recently, a large cluster randomized controlled trial evaluating TCM in the UK (STARS) reported a transient small improvement in all children’s mental health, but a larger effect sustained over 30 months among children with poor mental health at baseline (Ford et al., 2019b). Although there were no changes in measures of teacher distress, burn out, and self‐efficacy (Hayes et al., 2020), teachers who attended TCM reported a positive impact on themselves as teachers (feeling calmer, more confident and in control) as well as positive impacts on their pupils and teacher‐parent relationships (Allen et al., 2020).

Although these studies suggest TCM may be beneficial to teachers and children, such benefits will only occur, and be sustained, if teachers find the TCM approach acceptable and implementable; however, few studies have considered this within the UK. In North Wales and England (UK), Hutchings et al. (2007) and Marlow et al. (2015) conducted interviews with teachers and reported high levels of satisfaction with content and delivery. Although these initial findings suggest the TCM approach may be acceptable, both studies were relatively small, and Hutchings et al. (2007) only considered the acceptability of the 5‐day version of TCM, rather than the full 6‐day programme. Thus, there is still a need for an in‐depth consideration of acceptability and implementation of the TCM programme within the UK.

Both acceptability and an understanding of the factors influencing implementation are key components to the successful adoption and sustained provision of any novel intervention (Hagermoser Sanetti & Collier‐Meek, 2019). As this may vary across contexts, it is important to understand whether teachers in the UK consider TCM relevant to their training needs and whether they are able to implement their learning in the classroom. Exploring teachers’ experiences may identify key factors for successful implementation, that if overlooked might contribute to the research‐to‐practice gap within education (Hagermoser Sanetti & Collier‐Meek, 2019).

The current study examines the acceptability of the full 6‐day TCM programme and perceived barriers/facilitators to implementation in the English (UK) primary/elementary school context for children aged 4–11 years by reporting on the findings from the process evaluation of the STARS trial (Hansford et al., 2015).

## Materials and methods

STARS was a cluster randomized controlled trial evaluating the effectiveness and cost‐effectiveness of the TCM programme conducted in Devon (UK) compared to teaching as usual between 2012 and 2017 (Ford et al., 2019b). Eighty primary schools were offered the opportunity to send one teacher from a reception to year 4 class (teaching 4‐ to 9‐year‐olds) to attend the TCM programme. Teachers from schools randomized to the intervention (*n* = 40) attended training in their first year of the trial, those in the control schools (*n* = 40) attended in their second year of the trial.

The current study reports on the findings from the process evaluation of the above trial. Materials and methods relevant to the current study are summarized below and described in greater detail in an earlier paper (Allen et al., 2020).

### The IY TCM programme

The IY TCM programme focuses on building positive relationships with students; preventing problem behaviour; teacher attention, coaching and praise; motivating children through incentives; decreasing inappropriate behaviour; and emotional regulation, social skills, and problem solving (Webster‐Stratton et al., 2011). The six sessions ran between November and April within one academic year and were delivered by trained IY TCM group leaders (GLs). TCM groups included between 8 and 12 teachers and measures were undertaken to ensure fidelity to the model (Ford et al., 2019a). Schools were provided with funding for supply cover to release teachers to attend and were not charged course fees.

### Participants and data collection

Teachers were invited via email to take part in a focus group 2 months after the completion of their TCM training. Teachers were offered a telephone interview if they were unable to attend this focus group. Both were described as an opportunity to explore their experiences and views of TCM, both positive and negative, and were conducted by a trained researcher‐facilitator.

Each focus group ran for approximately one and a half hours and a topic guide was used to facilitate the discussion. The topic guide was developed by the research team as part of the process evaluation for the STARS trial (Hansford et al., 2015) and included questions that had arisen due to the ongoing relationships with teachers taking part in the study. The topic guide focused on teachers’ perceptions of TCM, what factors might impact the use and implementation of TCM and what impact they felt it had had/not had (in terms of themselves, children, parents, and wider school environment; reported in Allen et al., 2020). Telephone interviews were of a similar duration and followed a parallel topic guide. Focus groups and interviews were audio recorded for transcription and analysis. All participating teachers received a £10 gift voucher as a thank you for their time.

Data saturation was reached after five focus groups. Of the 47 primary school teachers who attended one of the first five TCM programmes run as part of the STARS trial, 44 took part in either a focus group (*n* = 31) or telephone interview (*n* = 13). The three teachers who did not participate in a focus group or interview declined to take part due to bereavement (*n* = 1) or maternity leave (*n* = 2).

Participating teachers were similar for both methods, most were: female (77% both groups); in their thirties (mean 34.6 years focus groups, 30.3 years interviews); worked full time (94%, 100%); and the mean length of their teaching career was between 6 and 7 years. Three teachers had leadership roles (all of whom attended focus groups) and two were newly qualified (one in a focus group and one in an interview). The mean proportion of children with Special Educational Needs or Disabilities (SEND) in the teachers’ classes was similar for focus groups (22.0%) compared to interviews (24.7%), but 65% of the focus group teachers were teaching younger children and 61% had full time classroom support compared to 31% and 54% of the teachers who were interviewed. Participating teachers attended a similar number of TCM sessions (mean attendance 5.7 sessions focus groups, 5.4 interviews), with only one teacher (in the interview group) having attended fewer than half of the six sessions.

### Analysis

LH led the qualitative data analysis, supported by KA, AP, and OM. Three of these researchers (LH, KA, and AP) were working full‐time on the STARS trial and one of the researchers was working on a number of different research projects (OM). LH had substantial qualitative research experience and ensured the rest of the team were adequately trained in qualitative analysis, providing ongoing supervision throughout the process. Data were managed using Nvivo 11 qualitative data analysis software; QSR International Pty Ltd. Version 11, 2015.

Thematic Analysis (Braun & Clarke, 2006) was employed to explore the data, and a coding scheme was jointly devised that was primarily guided by the research questions but also allowed for a more inductive analysis whereby additional themes extracted by researchers during the data analysis process could be included. A Framework approach (Richie & Lewis, 2008) was used to organize the data, within which categories were further refined and summarized and links and patterns between the resultant themes were explored.

To ensure rigour, the team held regular meetings throughout the data analysis process to check for data saturation (i.e. where no new themes are being discovered) and to identify, agree, and refine emerging themes. Any disagreements were discussed as a team and resolved.

## Results

Three main themes were identified: (1) acceptability of TCM; (2) facilitators to implementation; and (3) barriers to implementation (see Table 1).

**Table 1:** Themes and sub‐themes

Category	Theme
Acceptability of TCM	Structure
	Being in a group
	Delivery methods
	Group leadership
	Materials
	Time out of classroom
Facilitators to implementation	Theory
	Flexibility of strategies
Barriers to implementation	TCM programme content Challenging behaviour Appropriateness for older children Timing of programme
	School context
	Lack of consistency Lack of space and time Clash with existing school policies/behaviour management strategies Lack of support from senior leadership Pressure of being observed

### Acceptability of TCM

#### Structure

Teachers reported that the structure of the programme, i.e., 1 day per month for 6 months, worked well for them as it allowed time between each session to practise the suggested TCM strategies. The 6‐month duration was also important to teachers as it allowed the content to ‘evolve’ as the theory became more embedded in their practice, and helped facilitate the development of supportive relationships within the group, allowing discussions to deepen:

I think it’s nice having so many sessions, ‘cos when we all first sat down it’s a bit nerve wracking […]. Then as we got to know each other and it was a really nice group of people so you just relax a little bit more

#### Being in a group

Working and learning together with peers was deemed to be one of the most important aspects of TCM. Attending alongside teachers from a number of schools in diverse socio‐economic areas, varying year groups and with different levels of experience helped enrich discussions and provided a broader perspective from which teachers could reflect on their own practice:

We don’t get enough chances to talk to other schools about what they do […] it’s interesting to do that because sometimes you’re very much stuck in your own room. And when you hear about how other people work and what their schools are like […] you do take on some of their ideas

Teachers reported that discussing strategies that others had adopted, and ways in which they had adapted them, encouraged them to try new approaches.

Teachers liked the size of the groups (8–12 participants) and felt that if they had been larger it may have compromised how well they were able to get to know each other and how ‘safe’ they felt in openly sharing problems, experiences, and ideas. Feeling ‘safe’ within the group helped teachers feel supported and respected and allowed them to share without fear of judgement and so address issues in a positive and proactive manner:

For me it was like therapy for teachers […] having the time to come away from the classroom and realise that those little things that really bug you on a day to day basis everyone feels the same and it’s ok to get, you know, to feel at times frustrated […] just be reminded of the strategies and ways to deal with it, and that it’s OK, was really powerful for me and I went back to school each time for that next sort of few weeks feeling really great

This non‐judgemental environment was important to teachers who reported that learning alongside attendees from different schools allowed them to talk freely about any difficulties they were experiencing within their school context.

#### Delivery methods

Teachers liked the way in which the course was delivered, enjoying the modelling, rehearsing, practicing, and reflecting cycle of learning:

Behaviour policies from schools don't generally talk about all of those little positive things that you do as a teacher, they go right in with the sanctions […] being able to go into school, put those things into practice, do a bit of trialling, see what worked, see what didn't and then going back and discussing it again was actually really, really beneficial

Teachers believed seeing strategies modelled through video clips and role play helped to deepen their understanding of different behaviour management techniques and gave them the confidence to try them back in the classroom.

Role play was however one aspect of the delivery methods that prompted a mixed response from teachers in terms of acceptability. Some experienced it as uncomfortable and ‘contrived’, while others thought that, although uncomfortable at first, it helped them to understand situations from the child’s point of view:

I think they also made you think a lot about the child’s perspective ‘cos we had to do the role play, which to us felt really awkward, but it did make you really stop and think about how the child when you are talking to them and are responding to them what they are feeling like

#### Group leadership

Teachers reported that the GLs’ approach was key in helping to create a positive learning environment. Most teachers reported the GLs were welcoming, friendly, open, and supportive, and this was a crucial element of the programme:

There was a lot of humour but also they didn’t ever make you, they didn’t put you in a position where you felt awkward or uncomfortable, you were invited to share but not forced to and I think that is quite important

Teachers commented the GLs recognized them as experienced teachers and actively encouraged them to value and support each other, creating a relaxed space where they did not feel judged or patronized. Teachers appreciated the GLs’ flexibility to the needs of the group while still delivering the course content.

Most teachers liked the pace of the GLs’ delivery and the balance between leaders’ input and group discussion. However, a few teachers experienced the GLs’ style of delivery as patronizing, too fixed, and unresponsive, lacking the flexibility to respond to the needs of the group. These teachers thought their GLs were ‘not open to new ideas’ and commented the course failed to teach them anything new. However, despite this, most teachers still reported a positive impact from the course. One teacher who initially thought the course was ‘too basic’ reflected that, in retrospect, they understood the need for the initial focus on the foundational principles:

The first few sessions I felt were kind of like behaviour management 101 and [I] thought well, been doing this for many moons and I thought a lot of it was very, very basic […] Looking at it at the end, overall I can understand why there was an emphasis on that bit because it all sort of falls into perspective as to what is most important

#### Materials

Teachers’ views on the TCM materials provided during the programme were mixed. For instance, while some found the IY TCM textbook ‘daunting’ and difficult to read due to lack of time, others reported that it was useful to ‘dip in and out of’ and helpful when discussing strategies with parents and colleagues outside the TCM programme. One teacher reported it helped them to justify their time out of the classroom:

It was really useful though to show senior leadership team, and say look this the level that this course is going to be working at

The video clips, used throughout the course to demonstrate various TCM strategies, also received a mixed response. Teachers commented while they could be useful as a discussion stimulus, they were extremely outdated, too ‘Americanized’, and depicted much smaller class sizes than those typical in UK primary schools.

Teachers were much more positive about the handouts and ‘buzz’ documents, which they received at the end of each TCM session, describing them as useful prompts summarizing the key points to focus on between sessions.

Teachers thought some IY materials required adaptation for their classroom environment and this would be easier if they were provided electronically. IY produce a range of resources (e.g. puppets); however, teachers reported these were often too expensive and difficult to source.

#### Time out of classroom

Teachers valued being given time away from the classroom for reflection on and discussion about their practice:

I think what’s really helped me with the course is having that opportunity to step back […] for the day and reflect and actually think “oh yes, maybe I am getting a little bit negative with the behaviour or always maybe looking at the wrong thing” and that day having out of class helps you to re‐evaluate and think about the good things and you come back and you feel all inspired to try something else

This was a strong, overarching theme, with teachers describing being allowed space to re‐evaluate their practice away from the daily stresses and demands of the classroom as pivotal in changing their practice.

### Facilitators to implementation

#### Theory

Understanding the theory underpinning the TCM strategies motivated teachers to implement them. Teachers reported that the theory provided them with a more informed and structured approach to behaviour management in which they reflected on what they were doing, what the child was doing, and why”.

really understanding where those strategies come from as opposed to just going 'Oh yeah we'll give stickers because that's nice…It's really going through why you're saying what you're saying and what effect that that's having so it's much more thought through

**Figure 1:**
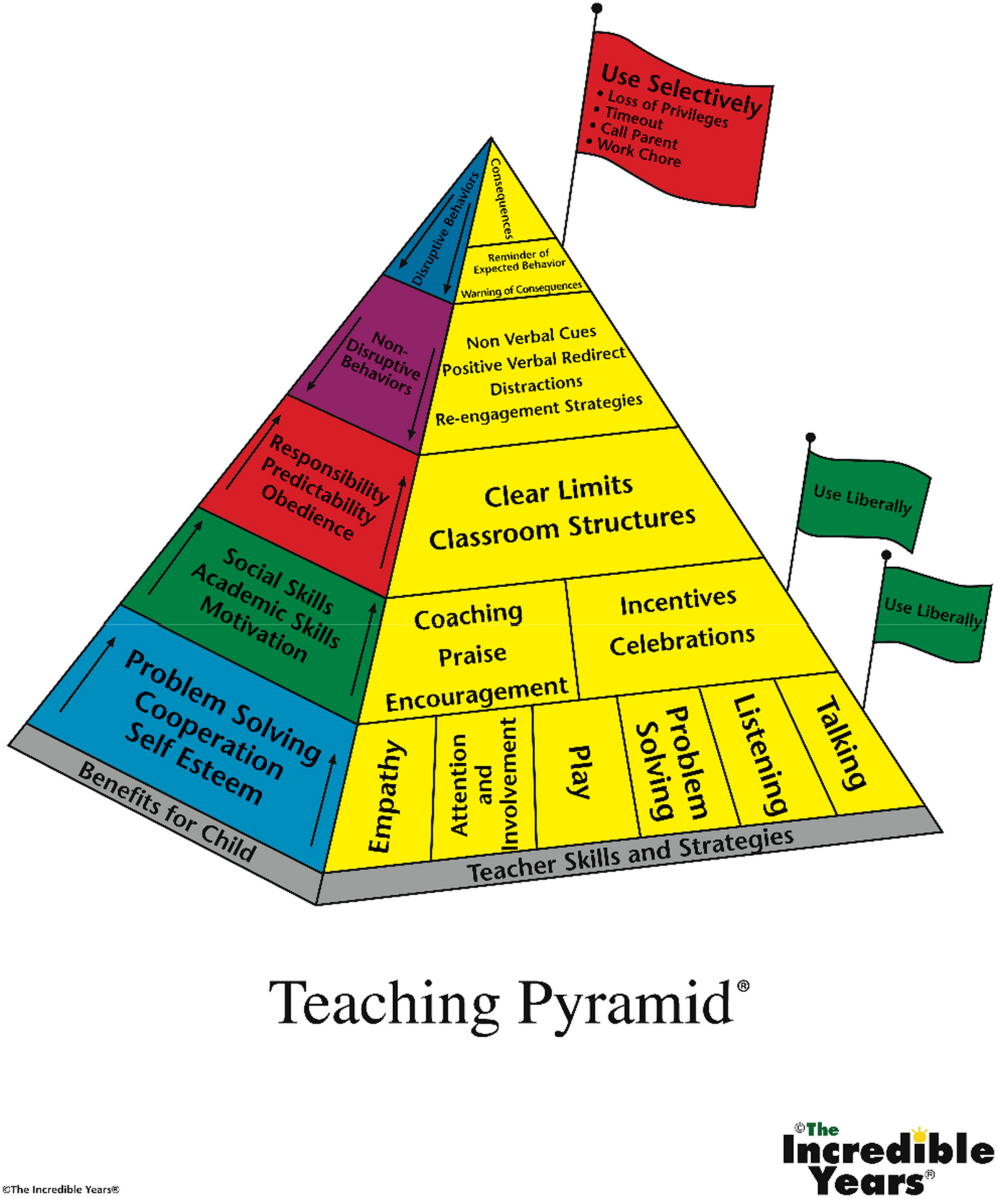
IY‐TCM Teaching Pyramid^®^ (property of The Incredible Years^®^, Inc. and Dr. Carolyn Webster‐Stratton and reproduced with their permission).

Teachers also really valued the IY pyramid (see Figure 1), which complements the underlying theories behind TCM, describing it as a useful way to explain TCM principles:

so it does just make you stop and think about the ways that you can organise your class and the ways that you can respond to the children before you reach that, that top of the pyramid

#### Flexibility of TCM strategies

Teachers reported the flexible nature of the TCM strategies was key to enabling implementation. Teachers valued the recognition that all teachers, teaching styles, children, classrooms, and schools are different and ‘no one strategy will work for everyone or forever’. Being able to tweak and adapt strategies to suit needs was deemed important.

### Barriers to implementation

#### TCM programme content

##### *Challenging* *behaviour*

Some teachers reported TCM did not address some of the more challenging behaviour they see in their classroom and they needed more in‐depth advice to help them manage the behaviour of these children and those with specific behavioural needs:

I’m just not convinced that [the course] has worked for my main offenders if you like, the ones who have ODD and ADHD and all of that kind of thing. I don’t think it’s really worked for them […] but for general classroom management it definitely has worked

##### *Appropriateness* *for older children*

One teacher felt TCM was more applicable to younger children (Key Stage (KS) 1, 4–6 year olds) and the strategies were less appropriate for the older children they teach in KS2 (7–11 year olds), which limited their implementation:

I had one of the older classes on the course so I have a year four, and a lot of the other people kind of had a year two or year one, foundation, and I understand that obviously a lot of it needs to be geared towards them, but there was little I could take away and use in an upper key stage two classroom

Others specifically thought two particular TCM strategies, the use of commentary and social coaching, did not work well with older children:

The only thing I haven’t really used is the like the commentary… I think as they’re a little bit older it’s not, I don’t feel it was as effective […] I don’t think it quite worked as well as it would if they were younger children

##### *Timing* *of programme*

Teachers reported the timing of TCM (i.e. running from November–April) was not ideal as it meant they were unable to put strategies into place at the beginning of the academic year:

I had to redo my rules after like in January when actually we should be setting the rules in September but it was just a different way of doing it

#### School context

##### *Lack* *of consistency*

A lack of consistency, at both classroom‐ and school‐level, was highlighted as one of the key barriers to implementing the TCM strategies, with teachers reporting that a ‘whole‐school’ approach was necessary to ensure proper implementation:

You need everybody on board really don’t you because if you’re going to be consistent with your behavioural management you need not just you to be doing, you need the other adults that you work with to be doing the same thing

Some teachers reported teaching assistants and other colleagues were too focused on ‘negatives’, finding it difficult to acknowledge positive behaviour in the classroom and adopt the new behaviour management techniques they were suggesting. Teachers identified this was often due to a lack of understanding which was difficult for them to address. Others reported when trying to use a strategy such as ignoring low level poor behaviour, there was a real danger that other staff, who did not understand the strategy, would ‘step in’ and therefore undermine it.

Many teachers suggested that training lunchtime assistants would be important for effective implementation, as they spend time with pupils and often use the ‘wrong’ methods to manage behaviour (e.g. reacting ‘angrily’). This in turn leads to the children being in the wrong frame of mind when they return to the classroom:

They don’t understand that they just can’t be like that with particular children because it does not get the best out of them […] it’s just going to make them more angry and they’re going to end up doing something else wrong which will get into a spiral of negativity

##### *Lack* *of space and time*

Teachers reported a lack of space and time as a barrier for implementing the strategies within their classroom. Some teachers felt they were always ‘racing to fit things in’, and others mentioned struggling to find time to share the TCM strategies and ideas with students, colleagues, and parents. This was deemed important not just for effectiveness but also to save ‘time and energy’ in the long term.

##### *Clash* *with existing school policies/behaviour management strategies*

The TCM approach and/or specific strategies clashing with existing school policies or systems were also identified as a barrier to implementation. Teachers understandably felt a pressure to follow existing school policies:

The only thing I haven’t really done is probably the timeout side of the programme and that’s more because we don’t use timeout at our school at all. Because we have a whole school behaviour policy I couldn’t really implement that just in my class

In contrast, some teachers had been encouraged by their senior leadership team (SLT) to develop new whole school behaviour plans after attending TCM, allowing them to implement strategies easily. In other cases, teachers had adapted TCM strategies so that they still worked within the school system. For example, one teacher reported using a lot more strategies before reaching the warning stage in the school’s behaviour policy:

I'm still following my behaviour policy but I've put so much more in place that I don't hit the first step of it yet […] so for instance our (school policy) is name on the board, three dots, headteacher […] but I'm getting to the point now where I'm putting in so much in before that I'm not needing to put a name on the board […] because that's personally something I don't agree with

##### *Lack* *of support from senior leadership*

Some teachers reported a lack of support from their SLT for adopting new approaches, hindering their ability to implement and disseminate the TCM strategies. Others mentioned they had specifically shared strategies with the SLT in order to gain approval to change behaviour management techniques.

Some teachers suggested feeding back information about TCM to their staff teams would be a good way to disseminate their learning and make implementation more effective and should be a mandatory part of the programme.

##### *Pressure* *of being observed*

During observations of their practice back in the classroom, teachers felt a pressure to conform to their school’s existing behaviour management strategies or be prepared to justify the alternative strategies they were using. This pressure to explicitly justify their use of strategies felt particularly strong when being observed by an OFSTED inspector:

If you’re being observed in‐house, actually you have more of a chance to argue why that’s the right strategy […] but with OFSTED you have to be seen to be doing things […] it’s just making sure that those strategies are very clear

Ignoring unwanted behaviour was a particular example of a strategy which teachers felt could be misconstrued as poor practice rather than understood to be part of a proactive behaviour management strategy.

## Discussion

On the whole, teachers who attended TCM experienced it as acceptable within their teaching context. They reported satisfaction with the programme structure, group learning environment, delivery methods, group leadership, and valued the opportunity to reflect on their practice outside the classroom environment. However, they were less satisfied with the video clips used throughout the programme, which they considered lacked cultural relevance, and reported that GLs’s style of delivery impacted their experiences of TCM. In relation to implementation, teachers highlighted understanding the theory behind TCM and the flexibility of the strategies as key facilitators. There were also several barriers to implementation, which included structural barriers such as conflicts with existing school‐behaviour policies and some dissatisfaction with elements of the TCM content, specifically strategies for dealing with the most challenging behaviour, and strategies appropriate to older children.

Teachers’ views on the acceptability of TCM mirror findings from previous, smaller, qualitative studies conducted both within the UK and other countries (Nye, 2017). Teachers liked the structure of TCM, allowing for time between sessions to implement the strategies in the classroom, the group element of the course and the seeing, practicing, and doing cycle of learning. Dissatisfaction with the video clips used during the programme, however, warrants some attention. Teachers thought the video clips were too ‘Americanized’, failing to reflect the behaviour in, and the size of, English classrooms. This is important as the video clips are used as a learning tool to encourage the adoption of specific teacher behaviours/strategies (Webster‐Stratton et al., 2011); however, such a tool is less likely to be effective if teachers are unable to relate to the setting or person modelling the behaviour/strategy. Our feasibility work (Marlow et al., 2015) and other qualitative studies (Baker‐Henningham & Walker, 2009) also highlighted that the video clips lack cultural relevance, with Nye (2017) arguing that this may partially explain why some teachers in Jamaica misinterpreted TCM strategies or had difficulty applying them (see Baker‐Henningham & Walker, 2009). Although teachers in the study questioned the acceptability of the video clips, this did not seem to impact on their ability to implement the strategies (Ford et al., 2019a), as GLs encouraged the teachers to see the value in the videos as discussion tools. More culturally relevant video clips may make this process easier though, requiring teachers to think less about how they might adapt the strategies to their classroom environment. Future research should consider whether this might be of benefit to teachers within English primary schools as well as other school contexts that might vary more substantially from the U.S. (where TCM was developed) in terms of class size and composition.

Teachers’ perceptions of TCM were influenced by how teachers experienced their GLs’ delivery style. Although most teachers found the GLs supportive, some perceived the leadership style as too rigid and unresponsive. In the U.S., researchers have similarly highlighted that GL ability to tailor TCM to meet teachers’ needs is a key facilitator for teacher uptake (Webster‐Stratton & McCoy, 2015; Webster‐Stratton et al., 2011). This may be more of a challenge for GL delivering TCM in school contexts outside of the U.S. The STARS trial ran over 5 years, with nine different TCM programmes delivered by different combinations of GLs (Ford et al., 2019a). Different groups inevitably have different dynamics, and the STARS trial worked hard to ensure GLs were sufficiently qualified, experienced, and adequately supervised (Ford et al., 2019a). These mixed comments highlight the need for supervision and support for GLs to allow them to reflect and respond to particular groups in particular contexts. This is important not only to ensure TCM is delivered with fidelity (Webster‐Stratton, 2004) but to deal with the inevitable tension between fidelity to the model and adaptation to the needs of particular groups. While some teachers were dissatisfied with the GLs approach, the fact that most teachers experienced TCM GLs as supportive suggests that this balance was mostly achieved.

The findings also shed light on the key facilitators to the implementation of TCM within English classrooms, which are likely to be applicable to other school contexts. Teachers described how understanding the theory behind the TCM strategies and the flexible nature of the strategies enabled them to implement TCM within the classroom. Teachers reported numerous ways they had adapted strategies to make them suitable for their classroom (Ford et al., 2019a). The relevance of underpinning theory and the flexibility of strategies is therefore something that should be recognized and highlighted by GLs delivering the programme, and also within initial teacher training. Providing additional support in adapting strategies to fit specific classroom environments through ongoing CPD has proven a useful way of harnessing these facilitators in Ireland (Davey & Egan, 2021), a strategy which could usefully be applied elsewhere.

Teachers did, however, perceive the programme as: (1) failing to help teachers deal with the behaviour of children who have SEND; (2) less useful for older children; and (3) starting too late in the academic year. In relation to children with SEND, this is an interesting finding given that Special Educational Needs Coordinators (SENCos) within participating STARS schools reported the TCM strategies were useful for supporting children with SEND (Nye et al., 2016). Furthermore, others have found TCM effective in reducing behaviour problems in children with more challenging behaviour (Nye, 2017). Some children have such severe difficulties they require additional support and adequate and timely specialist support for SEND is essential and sadly often lacking (Parker, Marlow, et al., 2016; Parker, Paget, Ford, & Gwernan‐Jones, 2016; Parker et al., 2018).

The second issue, that TCM was reported to be less useful for older children, has also been alluded to in other studies conducted in England (Nye, 2017) and is a common perception held in relation to the IY parenting programmes, although there is little empirical evidence to support this (Leijten et al., 2018). TCM was initially conceptualized as most appropriate for children aged 3–8 years old, but since then many primary schools adopting a whole‐school approach have successfully used TCM with teachers of 9‐12 year old children (Webster‐Stratton, 2013). No studies have suggested there is any difference in the impact of TCM on different age groups, suggesting TCM can indeed be useful for older children too. It is perhaps a question of emphasis and maybe reflects the composition of TCM groups in the study. Future work should test more homogenous groups of teachers in terms of the age group taught to see if this reduces the concern about how to work with older primary school children.

Teachers expressed a concern that the training started too late in the academic year and would have liked to be able to work consistently with their pupils from the outset. Given the experiential nature of TCM, ensuring teachers were fully trained by the beginning of the academic year would be difficult to achieve, as teachers need to be in the classroom during the training. However, these teachers’ comments provide an argument for including this type of programme in initial teacher training. Unfortunately, like many other countries teacher training in England does not involve any in‐depth modules on behaviour management, an issue that needs addressing (Carter, 2015; Perry, Booth, Owen, & Bower, 2019). Giving teachers these tools early on would enable them to be ready to deal with any class they subsequently have within their teaching career. Australia is good example of where this is already happening, with classroom management being a key component of initial teacher training (Perry et al., 2019).

The remaining barriers to implementation were all related to the school context; inconsistent behaviour management approaches among colleagues; a lack of time to implement and disseminate strategies; conflict with existing school policies; lack of support from SLT; and the fear that observers would misunderstand the behaviour management strategies applied. Sebastian, Herman, and Reinke (2019) have studied the part that contextual factors, such as school leadership, may play in moderating the impact of TCM on children. As others have already suggested, a whole school approach to TCM may overcome these barriers (Hutchings & Williams, 2017). Training all staff (teaching and non‐teaching) in TCM could: ensure consistency in behaviour management approaches; alleviate pressure on teachers to disseminate and explain their strategies to colleagues; and lead to school policies and systems that are consistent with and support the TCM approach. Studies report that whole school approaches may be useful when implemented effectively (De Noble, London, & El Baba, 2015); however, with a recent report suggesting they can add little to individual classroom management (Moore et al., 2019), such a programme should be rolled out within the context of careful evaluation. Important issues to be considered include the significant time commitment and costs of training. For this reason, TCM was trialled in its most basic form, with no additional coaching from GLs (Ford et al., 2019a). Additional coaching could become the remit of education support staff around children posing particular behaviour management challenges if TCM were more widely implemented. External coaching could be supplemented by school‐based peer support meetings to reflect on and develop practice. Any evaluation should include an economic component and careful assessment of pupil and teacher outcomes.

Our study adds to limited literature on teachers’ perceptions of the acceptability of the TCM programme and provides an in‐depth account of the facilitators and barriers to implementation as perceived by teachers within the English school context. Although some of these barriers and facilitators are likely to be specific to English schools, we believe others are likely to be applicable to other school contexts. Firstly, development of culturally appropriate video clips, and highlighting the adaptability of strategies for specific classroom contexts through flexible GL delivery or provision of additional coaching, is likely to be important in other countries outside of the U.S. (where TCM was developed) given international variations in classroom size and composition. Despite the similarities between the English and U.S. school context, teachers in our study highlighted these factors as important. Secondly, school‐level barriers to implementation are likely to be applicable to other contexts where TCM has only been delivered to individual teachers or classrooms rather than whole age groups or schools. Several countries have now trialled or implemented TCM across whole schools such as the U.S., Norway, Wales, and Jamacia (Aasheim, Reedtz, Handegård, Martinussen, & Mørch, 2019; Baker‐Henningham, Walker, Powell, & Gardner, 2009; Hutchings & Williams, 2017; Carolyn Webster‐Stratton & McCoy, 2015). Valuable lessons can be learned from these studies in terms of how this can be achieved and measures needed to sustain impacts, such as additional coaching (Aasheim, Fossum, Reedtz, Handegård, & Martinussen, 2020).

Although one of the larger studies of its kind, our study only provides the views of teachers within a specific geographical area (Devon, UK). Future research into TCM should include teachers from other areas, particularly those teaching in schools with catchment areas of high population density and ethnic diversity as well as gaining views of TCM from other key stakeholders, such as SLT, education support services, and parents. Future studies should explore whether training more teachers per school, or even all staff, would increase the impact of TCM on children and teachers.

### Conclusion

Most teachers participating in this study reported that the TCM programme was acceptable; however, some materials could be adapted to make the course more relevant to the UK context. Teachers reported that the theory and flexibility of the strategies enabled them to implement strategies within the classroom, but they also reported a number of barriers to implementation which, when taken into consideration, would improve scalability in the UK. We suggest that a ‘whole school’ approach to implementation may be one of the best ways to overcome these barriers.

## Conflicts of interest

All authors declare no conflict of interest.

## Author contributions

**Sarah Byford** (Conceptualization; Methodology; Supervision; Validation; Writing – review & editing) **Anna Price** (Data curation; Formal analysis; Investigation; Writing – review & editing) **Tamsin Ford** (Conceptualization; Funding acquisition; Methodology; Supervision; Validation; Writing – review & editing) **Brahm Norwich** (Conceptualization; Methodology; Supervision; Validation; Writing – review & editing) **Matthew Allwood** (Data curation; Formal analysis; Investigation; Writing – review & editing) **Lorraine Hansford** (Conceptualization; Data curation; Formal analysis; Investigation; Writing – original draft; Writing – review & editing) **Kate Allen** (Data curation; Formal analysis; Investigation; Writing – original draft; Writing – review & editing) **Bryony Longdon** (Investigation; Project administration; Writing – review & editing) **Rachel Hayes** (Formal analysis; Investigation; Supervision; Validation; Writing – review & editing).

## Funding information

This work was supported by the National Institute for Health Research Public Health Research Programme (project number 10/3006/07) and the National Institute for Health Research (NIHR) Collaboration for Leadership in Applied Health Research and Care South West Peninsula. These funders had no role in study design, data collection, data analysis, interpretation of data, or writing of the paper. The views and opinions expressed therein are those of the authors and do not necessarily reflect those of the NIHR Public Health Research Programme, NIHR, NHS, or the Department of Health and Social Care.

## Data Availability

Ethical approval for the current study did not grant the sharing of data and, therefore, research data are not shared.
